# A “conversion‐deterioration‐double mutation” theory for the evolution and progression of colorectal cancer

**DOI:** 10.1002/cam4.4637

**Published:** 2022-03-13

**Authors:** Rui Wang, Zhaopeng Yan

**Affiliations:** ^1^ Department of Critical Care Medicine Shengjing Hospital of China Medical University Shenyang China; ^2^ Department of General Surgery Shengjing Hospital of China Medical University Shenyang China

**Keywords:** aberrant differentiation, cancer evolution, cancer stem cell, colorectal cancer, differentiation, metastasis, reprogramming, stem cell, tumor heterogeneity

## Abstract

In this study, based on some clinical phenomena and recently published knowledge, we proposed our “conversion‐deterioration‐double mutation” theory, which provides a possible unifying explanation for the evolutionary process of colorectal cancer cells in the human body. In this theory, we proposed that there is a partial interconversion and a jump conversion relationship among normal colorectal epithelial cells, colorectal cancer cells, stem cells, and cancer stem cells (conversion). This conversion leads to tumor heterogeneity. We also proposed that well‐differentiated cancer cells converted from cancer stem cells have a more aggressive pattern than primary cancer cells (deterioration). The deterioration of primary cancer cells leads to differences in treatment responses and prognosis. Finally, we speculate a double mutation theory, indicating that for metastasis to occur, both mutations of cancer cells and mutations of target organs are needed and should match and meet. All these three points constitute the “conversion‐deterioration‐double mutation” theory.

## Graphical abstract

The conversion leads to tumor heterogeneity. The deterioration of primary cancer cells leads to differences in treatment responses and prognosis. The double mutation leads to different types of metastasis.

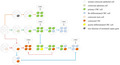

## 1 INTRODUCTION

The origin and progression of cancer involve multiple genes, multiple steps, and complicated processes. The complexity of these processes leads to a phenomenon known as tumor heterogeneity—the simultaneous presence of many different subclones of cancer cells within tumors. Cancer cells within a subclone have morphological and phenotypic profiles (incorporating cellular morphology, gene expression, metabolism, motility, proliferation, and metastatic potential) distinct from cells within other subclones.^1^ Tumor heterogeneity indicates that subclones within a single tumor can express different cancer biological behaviors, different responses to therapy and are among the main reasons for treatment failure among cancer patients. For a precise treatment purpose, studying how tumor heterogeneity arises during cancer progression and evolution is important to classify cancer subtypes so that better treatment strategies can be developed.

Currently, heterogeneity is explained by several models: the cancer stem cell (CSC) model, the clonal evolution model, and the plastic CSC model. In the CSC model, the cancer stem cell model proposes that tumors are organized hierarchically similar to normal tissue with rare multipotent and immortal CSCs at the top of the hierarchy, and transient, terminally differentiated non‐stem cancer cells form the bulk of the tumor.^2^, ^3^, ^4^ The clonal evolution model, also called the stochastic model, was first proposed in 1976 by Peter Nowell. In the clonal evolution model, tumor development is a Darwinian process driven by the accumulation of spontaneous genetic or epigenetic mutations, followed by successive selection of clones. Tumors develop from a single mutant cell, and this cell accumulates additional mutations as cancer progresses. These changes give rise to additional subpopulations, and each of these can divide and evolve further. This heterogeneity may give rise to subclones that have an evolutionary advantage over others within the tumor environment, and these subclones may become dominant within the tumor over time.^5^, ^6^, ^7^, ^8^ In the plastic CSC model, tumor heterogeneity result from the hierarchical organization of phenotypic cell states. However, in contrast to the CSC model, it is proposed that tumor populations behave in a dynamic fashion in which CSCs can differentiate into more mature progeny, and that differentiated cells may “de‐differentiate” back into stem‐like cells.^9^ These models can be validated in certain scenarios; however, no single model can comprehensively explain the heterogeneity and the evolution of cancer. Additionally, rare clinical phenomena that cannot be explained by existing models exist.

Colorectal cancer (CRC) is one of the most common cancers worldwide and reported having heterogeneity and metastatic ability.^10^ Metastasis is the major cause of cancer‐related death. Tumor heterogeneity and metastasis together make the management of CRC complicated. In this article, we propose a “conversion‐deterioration‐double mutation” theory to explain the evolutionary process, heterogeneity, and metastasis of CRC. Our “conversion‐deterioration‐double mutation” theory has three key points.

1. “Conversion”: There is a partial bidirectional interconversion relationship and a jump conversion relationship among normal colorectal epithelial cells, stem cells, non‐CSC colorectal cancer cells, and CSCs.
2. “Deterioration”: The differentiated daughter cells of CSCs are different from the previously differentiated cancer cells and are more aggressive as they can selectively inherit some features of CSCs based on the microenvironment stress.
3. “Double mutation”: For metastasis to occur, both mutations of cancer cells and mutations of target organs are needed and should match and meet.

## 2 PARTIAL INTERCONVERSION AND JUMP CONVERSION

**FIGURE 1:**
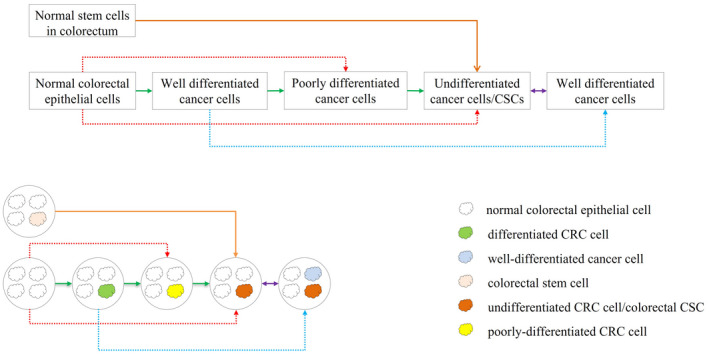
The partial interconversion and jump conversion relationships among normal epithelial cells, stem cells, non‐CSC CRC cells, and CSCs. In this figure, the solid line indicates a high possibility. The dotted line indicates a low possibility. (1) The orange arrow showed that the normal stem cells could convert into undifferentiated cancer cells/CSCs by de‐differentiation (rare). (2) The green arrows showed that how normal colorectal epithelial cells can convert into other types of cancer cells step‐by‐step by de‐differentiation (common). (3) The red arrows showed that normal colorectal epithelial cells sometimes can jump‐convert into other types of cells compared with green line by de‐differentiation (rare). (4) The blue arrow showed that cancer cells could directly convert into another type of cancer cells by reprogramming (rare). (5) The purple arrow showed that CSCs can divide and produce well differentiated but different cancer cells by re‐differentiation. (common), while these well differentiated but different cancer cells can convert into CSCs by de‐differentiation (common)

Recently, a new type of model describing tumor heterogeneity has been proposed: the plastic CSC model. The plastic CSC model proposes that tumor populations behave in a dynamic fashion in which CSCs can differentiate into more mature progeny and differentiated cells may “de‐differentiate” back into stem‐like cells, a process collectively referred to as bidirectional interconversion.^9^ Based on this bidirectional interconversion phenomenon, we extended the theory and proposed a partial interconversion theory, as shown in Figure 1. Figure 1 shows five lines of how cancer cells can evolve. (1) Orange line: Normal stem cells in the colorectum can directly convert into CSCs. (2) Green line: Normal colorectal epithelial cells can undergo malignant transformation to convert into well‐differentiated cancer cells, which can then de‐differentiate and convert into poorly differentiated cancer cells, which can continue to de‐differentiate and convert into undifferentiated CRC cells/CSCs. (3) Red line: Sometimes normal colorectal epithelial cells jump the well‐differentiated cancer cell stage and directly convert into poorly differentiated cancer cells or undifferentiated CRC cells/CSCs. (4) Blue line: Well‐differentiated cancer cells convert into another well‐differentiated cancer cell by mechanisms such as metabolic reprogramming. (5) Purple line: Undifferentiated CRC cells/CSCs can differentiate into well‐differentiated cancer cells, while well‐differentiated cancer cells can de‐differentiate and convert back into CSCs. Overall, we have summarized and named these five lines as partial interconversions and jump conversions. We speculate that survival stress may be the driving force for arrows to proceed. Notably, the stronger the survival stress is, the more likely the arrow would jump to the distal box instead of the adjacent box, the lower possibility that it would be a successful jump (the incidence of a jump is positively related to survival stress, the success rate of jump is negatively related to survival stress).

In this section, well‐differentiated cancer cells refer to those mature cancer cells; poorly differentiated cancer cells refer to those immature cancer cells. In this article, the concept of CSCs is more prone to be similar or the same as the concept of undifferentiated cancer cells. CSCs and undifferentiated cancer cells share the most important biological feature of stem cells: primitive and pluripotent capacity, which enable daughter cells of undifferentiated cancer cells/CSCs can be diverse.

## 3 RE‐DIFFERENTIATION AND ABERRANT DIFFERENTIATION

Occasionally, CRC incorporates a component other than adenocarcinoma. For example, colorectal adenosquamous carcinoma contains both adenocarcinoma and squamous carcinoma,^11^, ^12^ and although these rare CRCs account for only 0.05%–0.2% of CRC cases, the overall, and CRC‐specific mortality rates of patients are higher than those of patients with other types of CRCs.^13^, ^14^ The presence of squamous carcinoma in colon adenosquamous carcinoma is intriguing because there is no squamous component in the normal colon. A similar phenomenon also occurs in primary alpha‐fetoprotein (AFP)‐producing colorectal cancers. Primary AFP‐producing colorectal cancer is extremely rare and causes confusion among clinicians. Hepatoid type is a subtype of primary AFP‐producing colorectal cancer, which has a striking morphological similarity to hepatocellular carcinoma. It has been widely accepted that AFP‐positive adenocarcinoma is believed to be malignant counterparts of fetal‐type alimentary tract epithelium. In this hypothesis article, based on the flowchart in Figure 1, we speculate these rare components originate from the re‐differentiation of CSCs. CSCs are extremely potent and pluripotent; therefore, their daughter cells have a wide range of potential to become any type of cancer cell, including squamous and hepatoid cancer cells.^15^

Here, we define some concepts for an easy understanding.

1. Primary cancer cells: cancer cells that are converted from normal colorectal epithelial cells, but not converted into CSCs jet. The primary cancer cells are differentiated cancer cells.
2. Re‐differentiation: the process by which CSCs split and give birth to differentiated daughter cells.
3. Re‐differentiated cancer cells: differentiated daughter cells of CSCs. These re‐differentiated cancer cells may have a difference from primary cancer cells in morphology, biological behavior, chemosensitivity, aggressiveness, and metabolic pattern.
4. Aberrant cancer cells: one subtype of re‐differentiated cancer cells, which have a notable difference from primary cancer cells in morphology, biological behavior, chemosensitivity, aggressiveness, and metabolic pattern. There are no obvious boundaries between re‐differentiated cancer cells and aberrant cancer cells.
5. Aberrant differentiation: the process in which CSCs produce aberrant cancer cells.

It has been reported that the tumor bulk of CRC contains a different component other than adenocarcinoma, indicating a more aggressive pattern, more metastatic potential, and poor prognosis.^12^, ^13^, ^14^, ^16^, ^17^ Therefore, we hypothesize that aberrant differentiation promotes tumor aggression and provides a survival advantage to certain subclones of aberrant cancer cells. As aberrant cancer cells are one subtype of re‐differentiated cancer cells, so we speculate that maybe it is a common phenomenon that re‐differentiated cancer cells are more aggressive than primary cancer cells. The aggressiveness may be inherited from certain features of CSCs. It should be noticed that re‐differentiation phenomenon is difficult to be recognized in clinical practice because they frequently do not have an obvious morphological change compared with primary cancer cells, making it difficult for pathologists to identify.

## 4 DOUBLE MUTATION THEORY

The mechanism underlying tumor metastasis remains unclear. In 1889, Stephen Paget, an English pathologist, proposed the seeds‐soil hypothesis, which treats spreading tumor cells as seeds and targets distal organs as soil.^18^ This seeds‐soil hypothesis proposed that metastasis depends on cross‐talk between selected cancer cells (the “seeds”) and specific organ microenvironments (the “soil”). In 1928, James Ewing challenged Paget's seed‐soil theory and proposed that metastatic dissemination occurs by purely mechanical factors that are a result of the anatomical structure of the vascular system.^19^ Here, we propose a novel double mutation theory to explain metastasis based on the pre‐existing seeds‐soil theory, which is an extension of the pre‐existing theories. Pre‐existing hypotheses focus mainly on the factors outside target organs, such as genetic or epigenetic changes in cancer cells or anatomic factors. The pre‐existing theory focuses on the dynamic changes of “seeds” but neglect the truce that the essence of “soil” may change, too. On our double mutation theory, we emphasize on both mutations in cancer cells and mutations in the tissue of target organs. The double mutation theory includes several principles as described below:

1. The process of metastasis requires double mutation: Mutation X and Mutation Y. Mutation X is the genetic or epigenetic alternations which enable primary cancer cells to adapt to the microenvironment of the target organ. Mutation Y is the genetic or epigenetic alternations in the tissue of target organs, which enable normal target organ tissues to change the microenvironment; the changed microenvironment makes the circulating tumor cells (CTCs) easy to adapt.
2. Mutations happen in the human body all the time. Mutation Y can cause changes in the microenvironment of target organs and gain the ability to accept circulating tumor cells. When there are no primary tumors, mutation Y is unintentional and harmless. When there are primary tumors producing CTCs, if foci with mutation Y have already rebuilt the microenvironment to allow organs and tissues to gain the ability to accept CTCs, metastasis can take place. It should be noticed that Mutation Y happens in tiny focus of target organ, the focus is composed of a small group of cells. Mutation Y does not happen in the entire target organ.
3. If Mutation X and Mutation Y match, metastasis will occur. A strong Mutation X, which indicates that cancer cells have an extremely strong migratory and adaptive ability, can neglect the influence of Mutation Y on metastasis. A weak Mutation X and weak Mutation Y lead to the migration of cancer cells in the target organ to maintain a dormant state until Mutation X or Mutation Y becomes strong.

## 5 THE “CONVERSION‐DETERIORATION‐DOUBLE MUTATION” HYPOTHESIS

Based on the discussion above, we propose our “conversion‐deterioration‐double mutation” theory, which includes three rules:

1. Conversion: Survival stress can drive differentiated primary cancer cells or stem cells to undergo a de‐differentiation process and convert into CSCs, and CSCs can be converted back into re‐differentiated cancer cells. The bidirectional interconversion between CSCs and re‐differentiated cancer cells is easier than primary cancer cells and CSCs, as re‐differentiated cancer cells inherit some features of CSCs. If the evolving time is adequate (patients will not be killed by CRC, so the cancer cells have adequate time to evolve), the tumor bulk would be constituted with CSCs and/or re‐differentiated cancer cells. Whether the cancer cells will maintain the CSC status or re‐differentiated cancer cell status is decided by survival stress. Sometimes, the evolution of cancer cells can jump, which is also driven by survival stress. The incidence of a jump is positively related to the level of survival stress, and the success rate is negatively related to the level of survival stress.
2. Deterioration: Re‐differentiated cancer cells can be quite different from primary cancer cells and express a more aggressive pattern and survival advantage as they inherit some of the features of CSCs. This difference from primary cancer cells can be reflected in any aspect, such as morphology, cancer biological behavior, response to therapy, metabolic pattern, or prognostic pattern. Aberrant cancer cells are an extreme example of re‐differentiated cancer cells.
3. Double mutation: The occurrence of cancer metastasis needs to meet the match of Mutation X and Mutation Y. CSCs or some subclones of re‐differentiated cancer cells (such as aberrant cancer cells) should be considered as strong Mutation X, while most well‐differentiated primary cancer cells should be considered as weak Mutation X. A strong Mutation X can neglect the effect of Mutation Y.

## 6 DISCUSSION

Previous clinical studies are prone to include as many cases as possible to avoid bias and make the research results more robust and authentic. Scientists have taken adequate advantage of these common and mundane clinical cases to find clinical experience; therefore, we assume that there are no significant issues we can find from regular cases, and we might discover something new by focusing on rare clinical cases. Therefore, in this manuscript, we are prone to explore clinical laws from rare cases, such as adenosquamous CRC, AFP‐producing CRC.

### 6.1 The significance of tumor biomarkers

Our hypothesis of re‐differentiation/aberrant differentiation is also supported by clinical phenomena involving tumor biomarkers, such as cancer antigen 125 (CA125) and AFP.

In early‐stage CRC, the levels of serum tumor biomarkers are usually within the normal range, but in late‐stage CRC, the levels of these biomarkers can become extremely high. For example, high serum levels of CA125, a component of the female reproductive tract and a classic tumor biomarker of ovarian cancer,^20^ are rare in early‐stage CRC. However, in cases of metastatic CRC involving the ovary or peritoneum, serum CA125 levels are elevated.^21^ CA125 can participate in cell‐to‐cell interactions that enable the metastasis of cancer cells. This is supported by evidence showing that CA125 binds selectively to mesothelin, a glycoprotein normally expressed by the mesothelial cells of the peritoneum.^22^ CA125 and mesothelin interactions are thought to provide the first step in tumor cell invasion of the peritoneum.^23^ We assume that there are three possible explanations for the elevated serum CA125 levels in metastatic CRC involving the ovary. (1) Genetic or epigenetic mutation in CRC cells causes the ability to secrete CA125; in this scenario, a single gene or set of genes is altered. (2) Metastatic CRC cells are reprogrammed by the ovarian microenvironment and begin to secrete CA125. (3) By de‐differentiation, CRC cells convert to CSCs, and CSCs then undergo re‐differentiation/aberrant differentiation to convert into ovarian‐cancer‐like CRC cells, similar to ovarian cancer cells that are capable of secreting CA125, in which the entire cell, rather than a single gene or a set of genes, is altered. For possibility 1 to be true, elevations in serum tumor biomarkers would be more random than those observed in the clinic (elevated serum CA125 levels would be observed in liver metastasis, lung metastasis, bone metastasis, and other tumors, while elevated levels of serum cancer antigen 19–9 (CA19‐9) and cancer antigen 724 (CA724) are observed in CRC metastasis to the ovary). However, elevated CA125 levels are specific to CRC metastasis to the ovary, which negates possibility 1. In clinical practice, there are CRC patients who have removed ovaries; therefore, there is no ovarian microenvironment to reprogram the metastatic cancer cells, but they can still get a high serum CA125 level at a late stage. This phenomenon negates possibility 2. Therefore, we propose possibility 3 to be correct, in which CSCs produce ovarian‐cancer‐like CRC cells. These ovarian‐cancer‐like CRC cells are better suited to the ovarian microenvironment and are likely to metastasize to the ovary. According to our theory, for CRC patients, an elevation in serum CA125 levels is a sign of re‐differentiation/aberrant differentiation.

AFP is usually elevated in patients with hepatocellular carcinoma and yolk sac tumors.^24^, ^25^ AFP is produced and leads to an increase in serum AFP level. AFP‐producing colorectal cancer is extremely rare. AFP‐producing CRC has poor differentiation and shows aggressive biological behavior and worse prognosis than traditional CRC.^26^ A high metastasis rate and liver metastasis are the main characteristics of AFP‐producing CRC at initial diagnosis,^27^ and synchronous distant metastasis is commonly observed.^26^ Hepatoid adenocarcinoma is the main pathological feature of alpha‐fetoprotein (AFP) CRC.^27^ In the view of our “conversion‐deterioration‐double mutation” theory, it can be explained that CSCs (regardless of origin from stem cells or de‐differentiation of CRC cells) undergo an aberrant differentiation process and produce hepatic‐cancer‐like CRC cells, and these cells share the feature of secreting AFP, thus expressing an AFP‐producing pattern. Morphologically, they look like hepatic cancer cells and can adapt to the liver microenvironment well; therefore, they are more prone to liver metastasis.

Notably, the elevated serum biomarkers indicate that CRC cells may have undergone re‐differentiation/aberrant differentiation and acquired the ability to metastasize to certain target organs; however, this did not mean that metastasis had already occurred. This is consistent with the observation that liver metastasis is not always present in patients with CRC with high serum levels of cancer embryonic antigen (CEA), a biomarker of CRC. Among patients with colorectal liver oligometastasis who undergo hepatic resection, a high preoperative serum level of CA19‐9, a biomarker of CRC and hepatobiliary cancer, is predictive of poor prognosis.^28^ According to our theory, a high preoperative serum CA19‐9 level indicates that re‐differentiation/aberrant differentiation has already occurred, and the re‐differentiated/aberrant differentiated CRC cells have already acquired the ability to metastasize to the liver (strong Mutation X). There may even be tiny metastatic foci that are too small to be detected by imaging, and although surgery can be performed to remove visible foci, these tiny metastatic foci cannot be removed surgically, leading to cancer relapse and a poor prognosis.

### 6.2 The role of chemotherapy based on our “conversion‐deterioration‐double mutation” theory

In our “conversion‐deterioration‐double mutation” theory, we speculate that primary cancer cells are mostly chemosensitive. It has been reported that drug‐sensitive colon CSCs can interconvert with drug‐resistant colon cancer cells.^29^ In addition, not all CSCs are drug‐resistant.^30^ Therefore, we speculate that CSCs originating from de‐differentiation or stem cells are chemosensitive at first. When under survival stress (such as chemotherapy), CSCs can convert into drug‐resistant CSCs, and then re‐differentiated/aberrant cancer cells from drug‐resistant CSCs can inherit the drug‐resistant ability, expressing a drug‐resistant tumor bulk pattern.

Based on this speculation, we hypothesized that the degree of differentiation is not a predictor of chemosensitivity. As the primary cancer cells, even in a poorly differentiated degree, do not indicate the drug‐resistance ability and should be chemosensitive. However, re‐differentiated cancer cells from drug‐resistant CSCs with drug‐resistant ability do not respond well to chemotherapy. If this speculation is true, we can assume that: Adjuvant therapy should be initiated as soon as possible after surgery. For certain patients, chemotherapy without surgery can be a curative treatment.

Why adjuvant therapy should be initiated as soon as possible after surgery? Although adjuvant chemotherapy is typically started only after recovery from surgery, there is no consensus on the optimal time of treatment initiation. The guidelines from the European Society of Medical Oncology suggest that adjuvant therapy should be initiated as soon as possible after surgery and, ideally, no later than 8 weeks after the operation,^31^ adjuvant colon cancer trials typically mandate the initiation of chemotherapy within 6–8 weeks of surgery, and this has become the accepted timing of treatment. One explanation for the immediate initiation of adjuvant therapy after surgery is that early chemotherapy would eliminate residual CRC cells if they were primary cancer cells. Delayed chemotherapy provides residual cancer cells with a window of time to convert into CSCs, and later chemotherapy will convert the CSCs into drug‐resistant CSCs, and drug‐resistant CSCs can split and give birth to re‐differentiated cancer cells, which are chemoresistant and underlie chemotherapy failure.

Why for certain patients, chemotherapy without surgery can be a curative treatment? If a CRC is entirely composed of primary cancer cells, which indicates the absence of CSCs, it should be chemosensitive. Chemotherapy eliminates the entire tumor bulk. This phenomenon can be seen in some preoperative neoadjuvant chemotherapy patients in which the tumor bulk disappeared after chemotherapy.

### 6.3 Our views on tumor relapse, tumor metastasis, prognosis, and the effect of immune cells according to our “conversion‐deterioration‐double mutation” theory

It is widely accepted that the degree of differentiation is not a predictor of prognosis for CRC. According to our “conversion‐deterioration‐double mutation” theory, some well‐differentiated CRC cells may not be primary cancer cells but re‐differentiated cancer cells. These re‐differentiated cancer cells are more aggressive than even poorly differentiated primary cancer cells; therefore, the determination of primary cancer cells or re‐differentiated cancer cells would decide the prognosis, instead of differentiation degree.

Oligometastasis is a concept proposed in 1995 that indicates that cancer metastasis can have intermediate metastatic potential. During this stage, surgery of a metastatic lesion results in a relatively good prognosis.^32^ The theory of oligometastasis suggests that after surgery, the presence of a small number of metastatic foci indicates a relatively good prognosis, whereas that of a large number of metastatic foci indicates a relatively poor prognosis.^33^

Circulating cancer cells (CTCs) are the result of epithelial–mesenchymal transition (EMT). In our theory, both primary cancer cells and CSCs/re‐differentiated cancer cells can undergo an EMT process and become CTCs. According to our theory, we consider that CTCs from primary cancer cells have a weak Mutation X, while CTCs from CSCs/re‐differentiated cancer cells have a strong Mutation X. As mentioned in the theory previously, cancer metastasis needs to meet the needs of Mutation X and Mutation Y. The CTCs/re‐differentiated cancer cells with strong Mutation X can undergo a mesenchymal–epithelial transition (MET) process and form a metastatic lesion. Those CTCs with weak Mutation X cannot dwell on target organ and form a metastatic lesion, when encounter a weak Mutation Y. The primary cancer cells have a weak Mutation X; therefore, the metastasis needs to rely on Mutation Y. However, Mutation Y is a random event; therefore, less Mutation Y foci in target organ lead to a smaller number of metastatic foci, which expresses an oligometastasis pattern and a relatively good prognosis. If CSCs or re‐differentiated cancer cells migrate to a target organ, as they have a strong Mutation X, they can adapt well to the microenvironment of the target organ and do not rely on Mutation Y as much as primary cancer cells do; therefore, they can dwell anywhere inside the target organ, which expresses a non‐oligometastasis pattern and a relatively poor prognosis.

**FIGURE 2:**
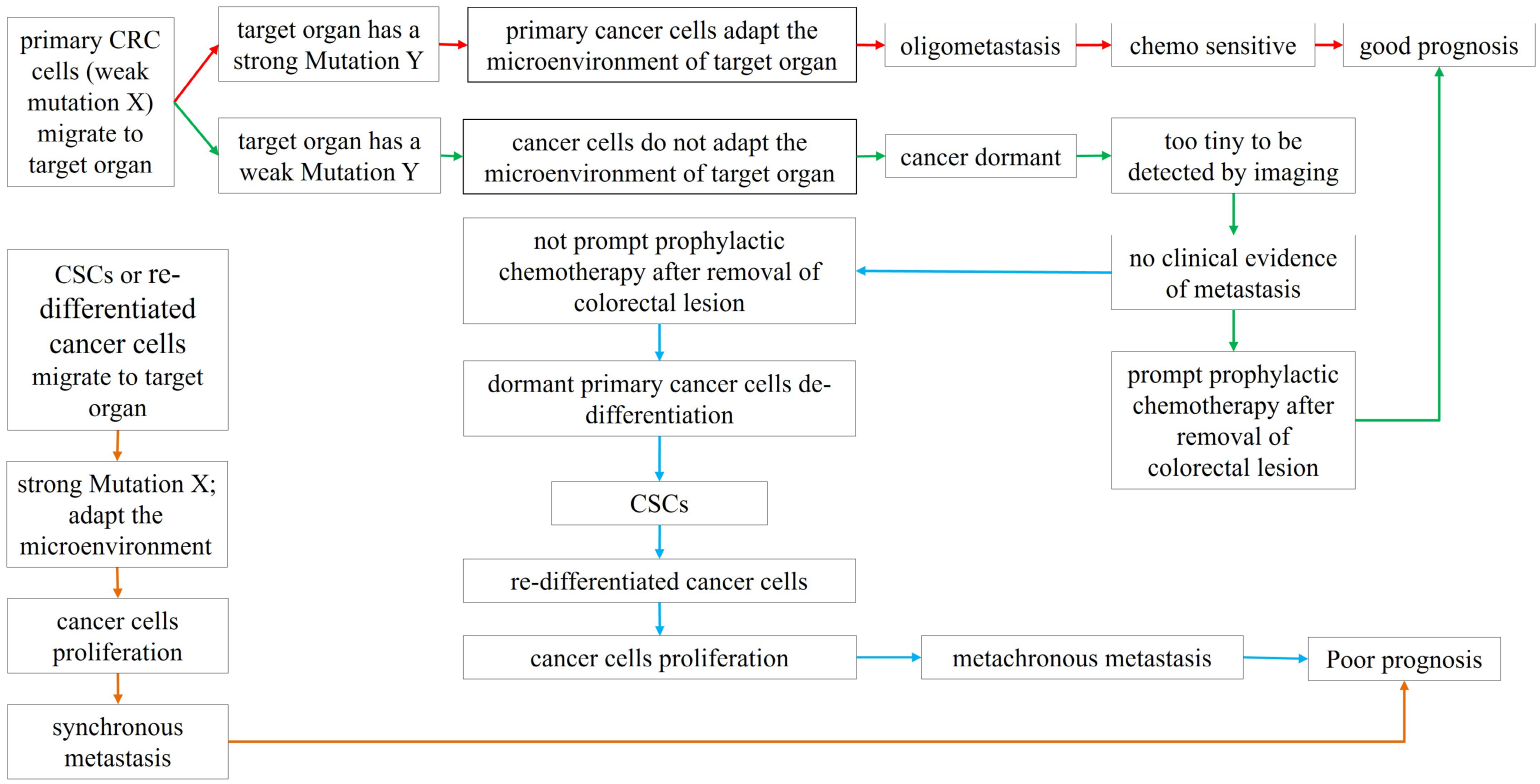
The metastasis and prognostic patterns of CRC. (1) In the red arrow approach, primary cancer cells migrate to the target organ, while there is a strong Mutation Y in the target organ, the primary cancer cells will adapt to the microenvironment well because of target organs' strong Mutation Y, and the metastatic cancer cells proliferate rapidly and form a metastatic lesion. As Mutation Y is a random event, which happens only small foci of target organ, the metastasis expresses an oligometastasis pattern. As they are primary cancer cells, they are chemosensitive and have a good prognosis after treatment. (2) Green arrow approach and blue arrow approach: primary cancer cells migrate to the target organ, while there is a weak Mutation Y in the target organ, the cancer cells do not adapt to the microenvironment well, the migrating cancer cells will not proliferate, and remain dormant. Because dormant cancer cells cannot maintain a large volume, the imaging examination cannot detect the metastatic lesion clinically, being considered as no evidence of metastasis. After removing the colorectal lesion, if prompt prophylactic chemotherapy is used, the dormant cancer cells will be killed, and the patients will be cured. If prompt prophylactic chemotherapy is not used, the dormant cancer cells will slowly undergo a de‐differentiation process, convert into CSCs, and succeed in re‐differentiated cancer cells, these CSCs and re‐differentiated cancer cells will adapt to the microenvironment of the target organ well and proliferate rapidly, expressing a metachronous metastasis pattern, which indicates a poor prognosis. (3) In the orange arrow approach, CSCs or re‐differentiated cancer cells migrate to the target organ, as CSCs or re‐differentiated cancer cells have a strong Mutation X, regardless of the status of Mutation Y. They can adapt to the microenvironment of the target organ and proliferate rapidly, expressing a synchronous metastasis pattern, which indicates a poor prognosis

Figure 2 shows the metastasis and prognostic patterns of CRC. There are several scenarios:

1. In the red arrow approach, primary cancer cells migrate to the target organ, while there is a strong Mutation Y in the target organ, the primary cancer cells will adapt to the microenvironment well because of target organs' strong Mutation Y, and the metastatic cancer cells proliferate rapidly and form a metastatic lesion. As Mutation Y is a random event, which happens only small foci of target organ, the metastasis expresses an oligometastasis pattern. As they are primary cancer cells, they are chemosensitive, and have a good prognosis after treatment.
2. Green arrow approach and blue arrow approach: primary cancer cells migrate to the target organ, while there is a weak Mutation Y in the target organ, the cancer cells do not adapt to the microenvironment well, the migrating cancer cells will not proliferate, and remain dormant. Because dormant cancer cells cannot maintain a large volume, the imaging examination cannot detect the metastatic lesion clinically, being considered as no evidence of metastasis. After removing the colorectal lesion, if prompt prophylactic chemotherapy is used, the dormant cancer cells will be killed, and the patients will be cured. If prompt prophylactic chemotherapy is not used, the dormant cancer cells will slowly undergo a de‐differentiation process, convert into CSCs, and succeed in re‐differentiated cancer cells, these CSCs and re‐differentiated cancer cells will adapt to the microenvironment of the target organ well and proliferate rapidly, expressing a metachronous metastasis pattern, which indicates a poor prognosis.
3. In the orange arrow approach, CSCs or re‐differentiated cancer cells migrate to the target organ, as CSCs or re‐differentiated cancer cells have a strong Mutation X, regardless of the status of Mutation Y. They can adapt to the microenvironment of the target organ and proliferate rapidly, expressing a synchronous metastasis pattern, which indicates a poor prognosis.

According to our theory, if CSCs and re‐differentiated cancer cells migrate to a target organ, they do not need a dormant stage but rapidly proliferate and form a metastatic lesion and are more prone to express a synchronous metastasis pattern. If primary cancer cells migrate to target organs, as Mutation Y is random event, they usually undergo a dormant stage to convert into CSCs and re‐differentiated cancer cells; therefore, more time is required to form the metastatic lesion and are more prone to express a metachronous metastasis pattern. Interestingly, patients with CRC and synchronous metastasis are more likely to have poorly differentiated tumors, lymphovascular invasion, advanced pathological tumor (T) and node (N) categories, and liver metastases than those with metachronous metastasis, consistent with our hypothesis.

CRC with isolated para‐aortic lymph node metastasis, well‐differentiated cancer cells, and low‐volume tumors (<two metastatic lymph nodes) indicate a good prognosis, but the reason why well‐differentiated cancer cells and low‐volume tumors indicate a good prognosis cannot be explained.^34^, ^35^, ^36^ According to our theory, well‐differentiated and low‐volume tumors are signs of primary cancer cell metastasis instead of CSCs or re‐differentiated cancer cells, which would indicate a good prognosis.

Regarding cancer immunity, previous theories suggest that lymphocytes have a direct inhibitory effect on cancer cells. According to our theory, we speculate that lymphocytes may have the ability to inhibit Mutation Y, which blocks cancer metastasis. In addition, there are reports that malignant tumors can recruit lymphocytes to promote cancer cells proliferation. Why do these lymphocytes promote the proliferation of cancer cells, instead of inhibiting the proliferation of cancer cells? According to our theory, one possible explanation is that these lymphocytes are trying to reduce the aggressiveness of the tumor, by reducing the survival stress of cancer cells. These lymphocytes secrete cytokines to assist cancer cells to proliferate, the assistance of these lymphocytes lowered the survival stress, and lower survival stress will delay the progression of aggressiveness of cancer cells and the origin of CSCs. So, indeed these lymphocytes are inhibiting cancer in the aspect of aggressiveness, instead of the proliferation of tumor.

### 6.4 The “conversion‐deterioration‐double mutation” theory vs. the consensus molecular subtypes

**FIGURE 3:**
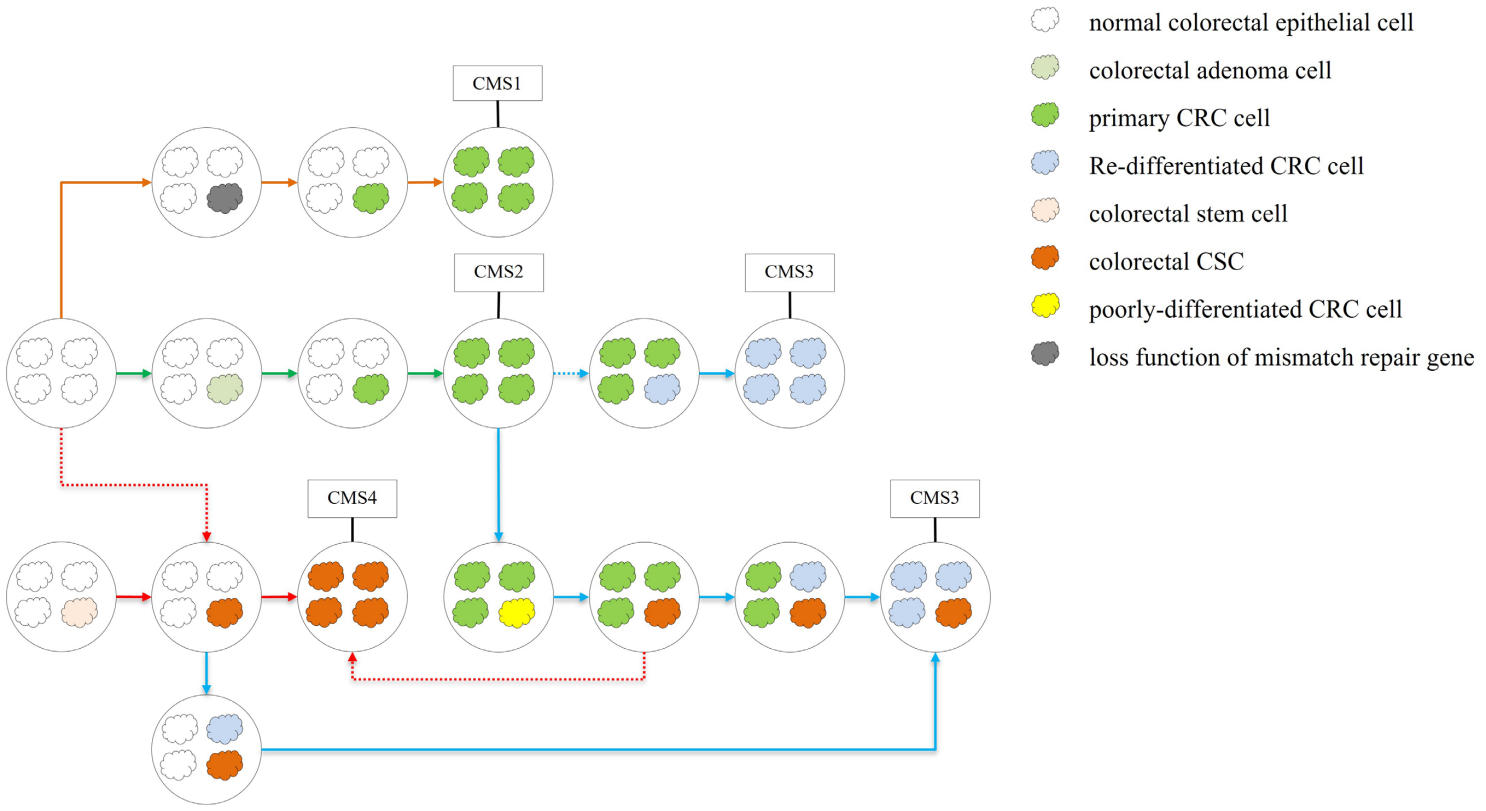
The evolution of CRC according to our “conversion‐deterioration‐double mutation” theory. For CMS1, CMS2, CMS3, CMS4, and mixed or indeterminate tumors, each of them can match a position in our model. In this figure, the solid line indicates a high possibility. The dotted line indicates a low possibility. This figure showed that CMS4 tumor and CMS3 tumor are the final stages of tumor progression. (1) The orange arrow showed how CMS1 tumors arise. Normal colorectal epithelial cells undergo a loss of function mutation of MMR genes and become CRC cells. (2) The green arrows showed how CMS2 tumors arise. Normal colorectal epithelial cells undergo an APC gene mutation and become colorectal adenoma cells. Then the colorectal adenoma cells undergo a p53 mutation to become CRC cells. (3) The blue arrow showed how CMS2 tumors convert into CMS3 tumors. The CMS2 cancer cells are differentiated into CSCs, and CSCs re‐differentiated and produce re‐differentiated cancer cells which have a different metabolic pattern. (4) The red arrow showed how CMS4 tumors arise. The normal stem cells in colorectal epithelium directly convert into CSCS

The consensus molecular subtype (CMS) model was proposed in 2015, indicating that CRC can be divided into four subtypes with distinguishing features.^37^ CMS1 possesses microsatellite instability and presents a higher histopathological grade. CMS2 has evident mutations in oncogenes and tumor suppressor genes. CMS3 possesses the features of evident metabolic dysregulation. CMS4 tended to be diagnosed at more advanced stages (III and IV) and stemness and displayed the worst overall survival. Figure 3 shows the evolution of CRC according to our “conversion‐deterioration‐double mutation” theory. Interestingly, we found that for CMS1, CMS2, CMS3, and CMS4 and mixed or indeterminate tumors, each of them can match a position in our theory.

#### 6.4.1 CMS1 tumor

According to our “conversion‐deterioration‐double mutation” theory, the de‐differentiation process and re‐differentiation process are survival stress‐driven. As shown in Figure 3, CRC with microsatellite instability indicates that mismatch repair (MMR) genes are out of function. This loss of MMR genes makes it easy for an epithelial cell to accumulate necessary mutations to become cancer cells and accumulate mutations which is a benefit for CRC survival. For CRC with microsatellite stable, it is not easy for them to accumulate mutations to survive as the MMR genes will correct the errors in DNA. So, microsatellites stable (functional mismatch repair genes) can be considered as a survival stress for carcinogenesis and cancer survival. For CRC with microsatellite instability, as there are fewer survival stresses compared with microsatellites stable CRC, they are prone to not undergo a de‐differentiation process and remain in a well‐differentiated status. This type of CRC with microsatellite instability coincides with the features of CMS1 tumors (presented with a higher histopathological grade).

#### 6.4.2 CMS2 tumor versus CMS3 tumor

CMS2 tumors have evident mutations in oncogenes and tumor suppressor genes, mainly involving APC and P53 genes. Mutations in the APC gene have a strong relationship with colorectal adenomas. The origin of the CMS2 tumor may have followed the classic adenocarcinoma–carcinoma model in the origin of CRC. According to our “conversion‐deterioration‐double mutation” theory, CMS2 tumors promote CRC from the process of normal epithelial cells to carcinoma cells (green arrow in Figure 3). After CMS2 tumor appears, the cancer cells in CMS2 tumor will be driven to undergo a de‐differentiation process under certain survival stress, poorly differentiated cancer cells will convert into CSCs, and CSCs have a re‐differentiation process to produce re‐differentiated cancer cells. The re‐differentiated cancer cells continuously proliferate until they occupy the entire tumor bulk (blue arrow). The biological behaviors, morphological, metabolic, and genetic and epigenetic features of re‐differentiated cancer cells could change significantly and express evident metabolic dysregulation. From the figure, we can conclude that the CMS2 tumor may be an intermediate stage of CMS3 tumor. Alternatively, CSM2 tumors and CMS3 tumors can be considered different stages of a single CRC. Whether cancer maintains a CMS2 status or a CMS3 status depends on whether the tumor is under survival stress. According to Figure 3, there are two other approaches for the production of CMS3 tumors.

#### 6.4.3 CMS4 tumor

According to CMS theory, CMS4 tumors have stemness, tend to be diagnosed at more advanced stages and display the worst overall survival. According to our theory, normal stem cells in colorectum can directly convert into CSCs, as the CSCs continuously proliferate, it can become a CSCs‐filled tumor. From the features of CSCs, we can conclude that this CSC‐filled tumor may have chemoresistance, strong invasion and migration ability, and poor overall survival. This CSC‐filled tumor shares the features of the CMS4 tumor and matches the position of the CMS4 tumor.

#### 6.4.4 Mixed or indeterminate tumors in the frame of the CMS model

As shown in the figure, some tumors in the middle stage in the boxes do not match a CMS subtype. We speculate that they match the mixed or indeterminate tumors of the CMS model.

In this section, we can conclude the CMS subtypes according to our “conversion‐deterioration‐double mutation” theory. It seems that the CMS model can be incorporated into our theory.

## 7 CONCLUSIONS AND FUTURE PERSPECTIVES

In this article, we propose a “conversion‐deterioration‐double mutation” theory for CRC that provides a possible unifying explanation for the evolutionary process of CRC cells in the human body, with the goal of enabling better clinical decisions and developing more accurate treatment strategies. In addition, we proposed some treatment strategies for CRC and speculations about cancer immunity (lymphocytes inhibit cancer de‐differentiation by reducing the survival stress of cancer cells). However, this theory requires additional supporting evidence.

There is an approach to verify our theory. In vitro, we can select CRC cell lines (group A) and create a disadvantaged environment for cancer cells (such as low titter chemo agent, which can be considered as survival stress), and we speculate that there should be undifferentiated cancer cells to be seen. Then we select the undifferentiated cancer cells to culture under a no survival stress environment, we speculate that the undifferentiated cancer cells can convert back into differentiated cancer cells (group B). Then we can compare the aggressiveness between group A and group B by Transwell assay and wound healing assay. For the double mutation theory, there is no ideal method to prove it now and is worth further studying.

## CONFLICT OF INTEREST

The authors declare that the research was conducted in the absence of any commercial or financial relationships that could be construed as a potential conflict of interest.

## AUTHOR CONTRIBUTIONS

Rui Wang: Original draft preparation, Funding acquisition. Zhaopeng Yan: Conceptualization, Methodology, Supervision, Review & Editing.

## Data Availability

Data sharing is not applicable to this article as no new data were created or analyzed in this study.
